# Intensive evaluation of radiation stability of phlogopite single crystals under high doses of γ-ray irradiation

**DOI:** 10.1039/c8ra08565j

**Published:** 2019-02-20

**Authors:** Honglong Wang, Yaping Sun, Jian Chu, Xu Wang, Ming Zhang

**Affiliations:** Institute of Materials, China Academy of Engineering Physics Jiangyou 621908 China wanghonglong915@163.com mz10001_mzhang@sina.com

## Abstract

The evaluation of radiation stability of clay is important for the disposal of high-level radioactive waste (HLRW). In this study, phlogopite single crystals were irradiated by Co-60 γ-rays in air at a dose rate of 3.254 kGy h^−1^ with doses up to 1000 kGy. Subsequently, the radiation stability and mechanism of radiation damage were explored by RS, FT-ATR, XRD, TGA, CA, and SEM techniques. In general, phlogopite single crystals show worthwhile radiation resistance toward their chemical structure but poor radiation stability toward their crystalline structure. Upon irradiation, their chemical structure changed slightly, while their crystalline structure varied obviously. For the 1000 kGy-irradiated sample, the interlayer space *d* of the (001) lattice plane increased by more than 1% with a value close to 0.13 Å, showing expansion. This could be mainly ascribed to H_2_O radiolysis and framework breakage: the former seems more important. These variations had a considerable impact on surface hydrophilicity, while they had marginal impacts on thermal stability and morphology: the effect on surface hydrophilicity is dose-dependent. A lower dose of irradiation sufficiently reduced the hydrophilicity, while a higher dose recovered the hydrophilicity. For instance, the CA increased from 14° to 28° with dose increases from 0 kGy to 200 kGy and then decreased to approximately 20° as the dose continued to increase to 1000 kGy. In general, the crystalline structure is more sensitive toward γ-ray irradiation and phlogopites could be regarded as poorly radiation-resistant. In this procedure, H_2_O radiolysis occupies a crucial role and seems to be the dominant factor. This finding is meaningful to evaluate the radiation stability of clay matrixes and to understand the microscopic property variations in clays used in practice when they are under irradiation.

## Abstract 2

Upon irradiation, the framework underwent breakage, H_2_O underwent radiolysis, and the radiolysis products reacted with the framework, expanding the lattice plane.

## Graphical abstract

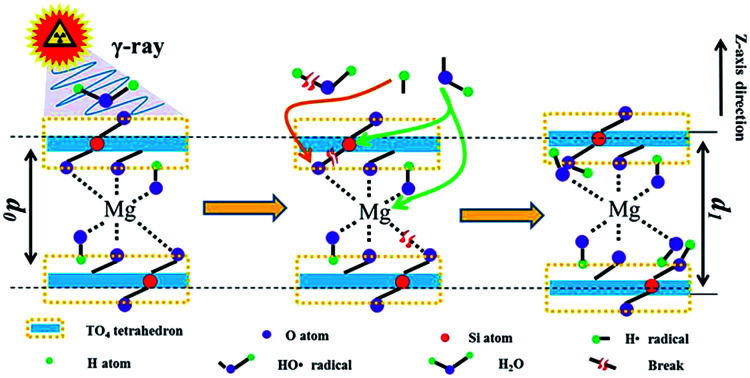

## 1. Introduction

Clay is a type of soil or mineral comprising several species such as montmorillonite, bentonite, talc, mica, *etc.*, and it is distributed widely in the world. The main component of clay is polysilicate such as aluminosilicate, alumina-magnesia silicate, and so on. In most cases, polysilicate possesses a layered structure and tetrahedron–octahedron–tetrahedron (T–O–T)^1^ or tetrahedron–octahedron (T–O)^2^ stacking along the *z* axis. The main framework of polysilicate is a tetrahedron composed of Si–O bonds. Except for tetrahedron, an octahedron exists and is composed of M–OH (M = Mg, Al, *etc.*) polar covalent bonds. Normally, partial Si atoms within the tetrahedron are substituted by Al atoms, yielding negative charges.^3^ In order to balance these negative charges, partial cations such as K^+^ or Na^+^ ions exist in the interlayer, leading to an uneven distribution of charges.^3^ In addition, a layered structure has numerous cavities trapping other species such as Fe^3+^ or Fe^2+^ ions, enhancing the uneven distribution of charges. In most of these cases, the interlayer only exudes the van der Waals force, resulting in the facile movement of the adjacent layers. Because of these characteristics, clay is ion-exchangeable, flame-resistant, and economically viable, and it has been widely used in the manufacture of adsorbents,^4^ flame-resistant materials, and functional materials. For instance, Sassi *et al.*^5^ simulated the retention behavior of Cs^+^ in phlogopites; Chen *et al.*^6^ sufficiently improved the flame resistance of poly(vinyl alcohol) by the addition of a small amount of montmorillonite.

Due to the low cost^7^ and excellent ion-exchange capability, clay is recommended as a backfill material to hinder radionuclide migration in the disposal of high-level radionuclide waste (HLRW).^8^ Except for the retention of radionuclides,^9,10^ it can uptake water and bear various irradiations such as γ/β-rays for radionuclide decay during its service duration.^11,12^ This irradiation can lead to H_2_O radiolysis and destroy the structure of the clay matrix.^13^ When the matrix structure is destroyed, its retention capabilities and mechanical properties can vary. Partial radionuclides might migrate to groundwater, inducing severe health issues or disorders.^14^ When used as a backfill material, it exhibits excellent retention capability and worthwhile radiation resistance.^12^ In reality, clay retention is the ultimate approach to reduce the dangers induced by HLRW to the ecosystem and its worthwhile radiation stability is imperative in ensuring the effectiveness of disposal. In this case, a sufficient evaluation of the radiation stability and the mechanism for investigating radiation damage within clay becomes important. Till date, numerous groups have carried out research for this purpose, including H_2_O radiolysis, ion exchange of metal ions, structural variations, *etc.* For instance, Allard and Calas^15^ and Gournis *et al.*^13^ discussed Fe^3+^ reduction as a result of H˙ radicals generated by H_2_O radiolysis; Lainé *et al.*^16^ and Fourdrin *et al.*^17^ discussed the mechanism of H_2_ generation under electron-beam irradiation; Holmboe *et al.*^18^ explored the retention behavior of Cs^+^ in γ-ray-irradiated bentonite; Pérez-Conesa *et al.*^19^ simulated the diffusion behavior of UO_2_^2+^ in montmorillonite; Lainé *et al.*^20^ explored the stability of H˙ radicals in talc; Brey *et al.*^21^ investigated the effect of environmental conditions such as H_2_O content and salt concentration on soil during the γ-ray irradiation process. The research in this field is comprehensive and cannot be completely cited.

Nevertheless, the main mechanism and degree of radiation damage were not clearly understood and evaluated due to the complexity of the material itself in addition to the environmental conditions. In most of the cases, clay is a composite with numerous impurities,^2^ even occupying 40 wt%.^22^ Simultaneously, H_2_O generally exists in the material or environmental conditions. During the irradiation procedure, the matrix, impurity, and H_2_O can generate numerous radicals. These radicals can interact with each other, leading a complex result. In addition, even the material's distribution location could affect the radiation effect. Finally, the mechanism of radiation damage within clay is difficult to be clearly understood, and the research in this field is challenging.^23^ Nevertheless, having a clear understanding of the mechanism of radiation damage within clay is meaningful for the disposal of HLRW,^23^ and it is also essential in the evaluation of the effect of nuclear accidents on the environment (*e.g.*, Fukushima Daiichi nuclear power plant accident).^9^ The research in this field is essential for the sustainable development of nuclear power.

Normally, the main property of clay depends on the matrix. Having a clear understanding of radiation damage within the matrix is imperative. To better understand and compare, partial influence factors should be eliminated and the system should become simpler. For instance, impurities (*e.g.*, oxides) should be completely wiped out and the amount of H_2_O should be reduced. Taking these factors into consideration, pure polysilicate is probably suitable for use since the matrix of clay is polysilicate in nature. Phlogopite—KMg_3_(AlSi_3_O_10_)(OH)_2_—single crystals are a pure polysilicate crystal attributed to the trioctahedral mica family with a typical “1 : 2” layered T–O–T structure. Two TO_4_ (T = 75% Si, 25% Al) tetrahedron sheets are interlinked by an MO_6_ (M = Mg, Fe, *etc.*) octahedron sheet. K^+^ and Na^+^ cations lie in the interlayer,^24^ and the O–H vector points along the *z* axis. In some cases, the OH group can be replaced by F, Cl, *etc.*^25^ Although phlogopite single crystals have not been recommended for the disposal of HLRW, their layered structure is similar to the matrix of clay, which is recommended for this purpose. Simultaneously, the content of impurities and H_2_O lying in this material is marginal. For its purity and low content of H_2_O, the effect of impurity and H_2_O on radiation damage can be disregarded. In this case, the obtained radiation effect could be mainly related to the matrix crystal, disregarding the complexity and facilitating understanding. Under these conditions, the radiation effect could be close to the radiation damage within the matrix of clay used in practice as their radiation defects are similar.^26^ Taking these factors into consideration, phlogopite single crystals are the ideal material to investigate the mechanism of radiation damage within the clay matrix. It should be noted that even though the content of H_2_O in phlogopite crystals is small, its radiolysis cannot be avoided; generally, there is marginal H_2_O lying in the interlayers, edges, or on the surface,^1^ as well as environmental condition.^10^

For clay used in the disposal of HLRW, irradiation mainly contains γ/β-rays. β-rays used for weaker penetration probably results in the increased radiolysis of the solvents such as H_2_O^20^ or damage to the impurities not existing in the clay matrix,^21^ and this destruction probably just exists on the sample surface. γ-rays used for stronger penetration could even penetrate the packing material.^27^ In this case, clay might have more opportunities to bear γ-ray irradiation, and the matrix and interior can be irradiated. In this case, the effect of γ-ray radiation seems more important and visible. In addition, for stronger penetration, γ-ray irradiation has been widely used to investigate the radiation effect and modification.^28–30^ Therefore, in this work, γ-ray radiation was used. It should be noted that even under γ-ray irradiation, the radiolysis of the solvent and damage to the impurities cannot be avoided. Simultaneously, the reaction of its products cannot be avoided.

In this work, phlogopite single crystals were irradiated by Co-60 γ-rays in air at a dose rate of 3.254 kGy h^−1^ with doses up to 1000 kGy. The selected absorbed dose—1000 kGy—could cover even the total irradiation level for 500 years during disposal^31^ and meet the demand for disposal of spent fuel in practice.^32^ After irradiation, radiation stability and mechanism were explored. The main objectives of this study were (1) to explore the radiation stability of phlogopite crystals; (2) to better understand the mechanism for damage formation; and (3) to promote the understanding of the mechanism of radiation damage within the matrix of clay used in practice, facilitating the evaluation of the effectiveness of clay used in HLRW disposal, which is of great significance. Generally, a crystalline structure is sensitive toward irradiation, and phlogopites can be regarded as having poor radiation resistance. Simultaneously, H_2_O radiolysis occupies a crucial role in radiation defects.

## 2. Experimental section

### 2.1 Materials

Optically transparent phlogopite – KMg_3_(AlSi_3_O_10_)(OH)_2_—single-crystal films were brought from the University of Cambridge, UK. Its accurate composition was analyzed and cited as sample (277) in the literature,^25^ containing marginal additional elements such as Na or Fe.

### 2.2 Sample preparation and irradiation

Prior to irradiation, the film (thickness: less than 200 μm) was dried in an oven at 65 °C for 5 h to remove the absorbed water. Then, the film was folded in an aluminum foil and irradiated by Co-60 γ-rays (Institute of Nuclear Physics and Chemistry, China Academy of Engineering Physics, Mianyang, China) in air at a dose rate of 3.254 kGy h^−1^ with doses up to 1000 kGy. After irradiation, the films were stored in air at room temperature before characterization.

### 2.3 Characterization

#### Raman spectrum (RS)

RS experiments were performed on a Nicolet Almega XR Instrument from 90 cm^−1^ to 1300 cm^−1^ at a spectrum resolution of 0.9 cm^−1^, a 532 nm laser source, and power of 4.5 mW.

#### Fourier-transform infrared (FT-IR) spectra

FT-ATR experiments were performed on a Bruker Tensor 27 FT-IR spectrometer from 400 to 4000 cm^−1^ with 32 scans per spectrum and spectrum resolution of 4 cm^−1^.

#### X-ray diffraction (XRD)

XRD measurements were performed on a D8 ADVANCE X-ray powder diffractometer using Cu K_α_ irradiation with *λ* = 0.15418 nm, voltage of 40 kV, and current of 40 mA. The scanning 2-theta (2*θ*) and step size values were set at 5–90 and 0.02°, respectively, and all the patterns were analyzed by the Jade 5 software.

#### Thermogravimetric analysis (TGA)

TGA experiments were performed on a NETZSCH STA 449 F3 instrument from 50 °C to 500 °C at a heating rate of 10 °C min^−1^ and argon flow of 50 mL min^−1^.

#### Contact angle (CA) analysis

Static contact angle experiments were carried out on an XG-CAM contact angle meter. A drop of 2 μL purified water was placed onto the sample surface; immediately, a picture was recorded by the camera. Then, the CA was calculated by means of software. Each sample was measured 5 times at different locations to obtain an average datum.^33,34^

#### Scanning electron microscope (SEM)

SEM measurements were performed on a Zeiss MERLIN Compact 14184 instrument (Germany) at an acceleration voltage of 8 kV. Prior to the experiments, a thin layer of gold was coated onto the sample surface.^35^

## 3. Results and discussion

### 3.1 Chemical structure analysis

**Fig. 1:**
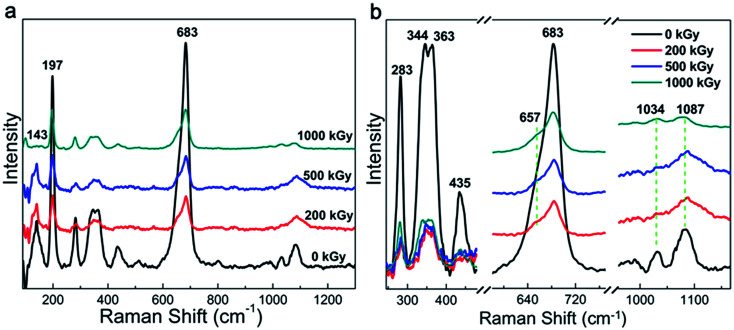
Raman spectra of phlogopite single crystals under γ-ray irradiation.

Raman and FT-IR spectra are widely used to characterize the chemical structure of a material. Fig. 1a shows the Raman patterns of the pristine and irradiated samples. All the spectra show 2 characteristic peaks at 197 and 683 cm^−1^ corresponding to the vibration of MgO_6_ octahedra and TO_4_ (T = Si, Al) tetrahedra, respectively.^36–38^ A partially refined structure can be observed in Fig. 1b and assigned in Table 1, which is related to the framework vibration observed in nature.^36–39^ As evident from the curves, no obvious changes in the peak pattern and sample position can be observed after irradiation. In other words, the Raman pattern indicates a slight variation in the framework. This phenomenon can be explained as follows. The Raman spectrum is efficient to characterize nonpolar vibrations.^40^ In this case, the spectra mainly reflect the natural Si–O vibration. Irradiation is effective to destroy chemical bonds.^34,35^ With regard to phlogopites, its TO_4_ tetrahedron structure is probably stable. This can be explained as follows. Although the Si–O or Al–O bonds can be destroyed leading to cleavage,^41^ broken Si or O atoms cannot leave their positions due to the presence of adjacent atoms. In this case, the main chemical bond species may vary slightly. In most of the cases, irradiation results in obvious chemical structure variations under the premise of the partial departure of atoms or the participation of additional species.^17,20,34,35,42,43^ As the main chemical bond species changed marginally after irradiation, the vibration of the framework also slightly changed. This assumption could probably explain the slight variations observed within the Raman spectra. Except for the peak pattern and position, the intensity might vary. Nevertheless, the affiliation is very complex, as shown in Table 1; for example, the peak at 1087 cm^−1^. It is difficult to affiliate this peak to an accurate or single vibration. Finally, it is difficult to quantificationally describe this variation. Except for the framework variation, a peak at 143 cm^−1^ shows obvious variations, which has complex affiliation, as shown in Table 1.^36,37^ Nevertheless, it probably contains OH vibrations.^37^ Under irradiation, the relative intensity of this band increased at 200 and 500 kGy and then decreased at 1000 kGy. This is very interesting. If we assume that this peak could reflect OH vibrations, then the content of the OH group increased at lower doses and subsequently declined at higher doses. This is informative but needs certification.

**Table 1:** Observed Raman vibrations and their phlogopite assignments

Peak position (cm^−1^)	Assignment
143	M2*x*-trans + O3-*xz*-trans + O1*x-*trans + (F, OH)*y*-trans
197	MO_6_ octahedron vibration (distortion)
283	M2*x*-trans + (F, OH)*y*-trans + O1–T–O1 bend or O–H–O vibration
344, 363	O2*x*-trans + M2*y*-trans, tetrahedral rot‖*z* + M2*y*-trans or O1–T–O1 bend + M2*z*-trans
435	M2–O3 stretch + T–O3*xz*-4trans
683	O3–T–O1 bend
1034	T–O3 stretch
1087	T–O1, T–O2 stretch and O1–T–O1 bend

Generally, the phlogopite framework changed marginally even under higher doses of γ-ray irradiation.

**Fig. 2:**
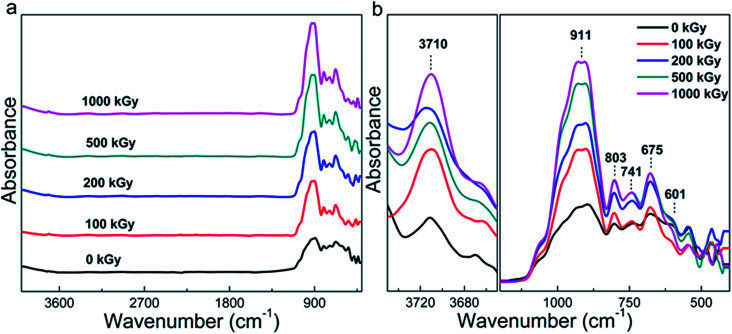
FT-ATR spectra of phlogopite single crystals under γ-ray irradiation.

Although the phlogopite framework changed marginally, asymmetric vibrations such as Mg–OH bonds probably changed obviously under irradiation. Fig. 2a shows the FT-ATR spectra of pristine and irradiated samples. For the irradiated sample, the spectra are similar to each other and the main peaks occupy less than 1200 cm^−1^. To clearly understand this, Fig. 2b shows the refined patterns in this region, and several peaks near 601, 675, 741, 803, and 911 cm^−1^ can be observed. Simultaneously, a peak at approximately 3710 cm^−1^ can also be observed. These peaks could be assigned as follows. The peaks at 601 and 675 cm^−1^ correspond to OH bending and O–Al–O vibration,^25^ respectively. The peaks at 741 and 803 cm^−1^ correspond to Al–O vibration and Si–O stretching,^25^ respectively. In addition, the peak at 911 cm^−1^ corresponds to Si–O–Al or Si–O vibration,^44^ and the peak at 3710 cm^−1^ corresponds to Mg_3_–OH vibration.

In most of the cases, Mg–OH bonds within the octahedron sheet can break easily because of the reaction 2OH → H_2_O + O.^17,45,46^ When Mg–OH bonds are broken, the octahedron structure becomes unstable,^47^ probably resulting in two results. One is that the octahedron deforms into a hexahedron.^38,48–50^ The other is that the octahedral Mg coordinates with tetrahedral O, forming extra Si–O–Mg bonds. Finally, more Si–O–Mg bonds near 375 cm^−1^ should exist in the irradiated samples.^25,44^ Hence, this peak could reflect variations within the octahedron related to the more visibly broken Mg–OH bonds. Nevertheless, this peak is very weak in nature^25^ and a region less than 400 cm^−1^ was not observed during the experimentation process. Therefore, it is difficult to conclude whether hydroxylation occurred or not during the irradiation procedure.

However, as evident from Fig. 2b, the peak at 911 cm^−1^ related to Si–O–Al vibration considerably changed, which is interesting. If sufficient Si–O–Mg bonds were not formed, in other words, H_2_O participated in the reaction. This would result in more Si–OH or Mg–OH bonds to be formed in the tetrahedron or octahedron sheets. When the Si–OH bond is formed, the amount of Si–O bonds (particularly related to non-bridge O) decreases. As the Si–O–Al bonds only exist in adjacent tetrahedra, its content probably cannot be affected by irradiation. Therefore, the content variation in Si–O–Al and Si–O bonds could reflect structural variations and the ratio of *I*_911_/*I*_803_ is considered. Here, *I*_911_ represents the intensity of Si–O–Al vibrations and *I*_803_ represents the intensity of Si–O stretching.

**Fig. 3:**
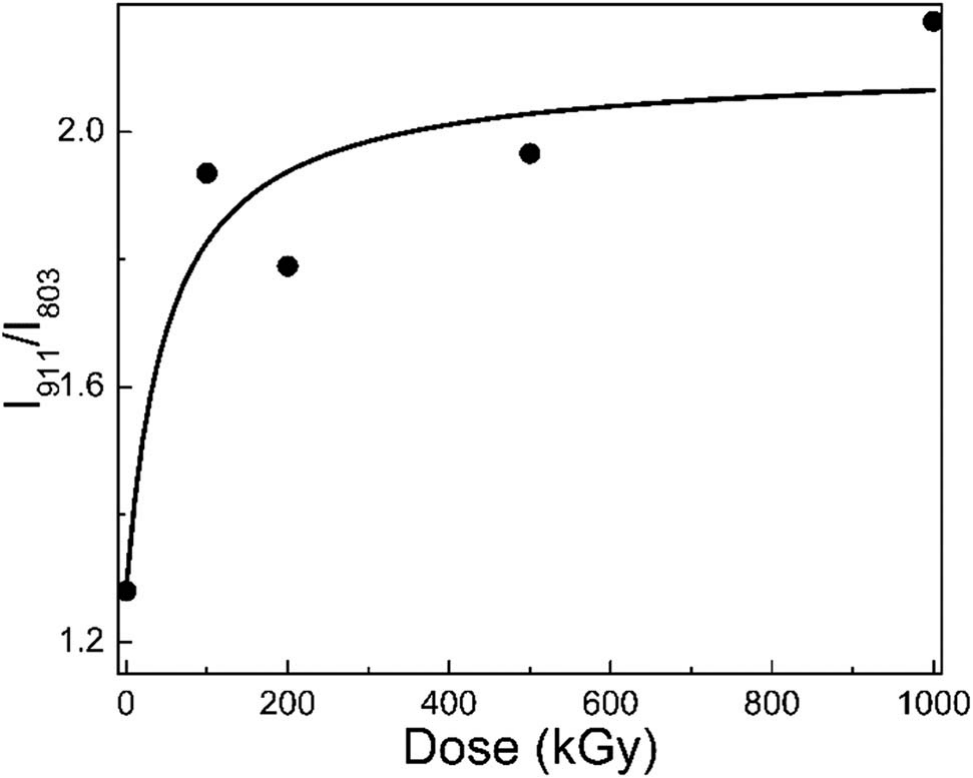
*I*_911_/*I*_803_ of phlogopite single crystals under γ-ray irradiation.

Fig. 3 shows the *I*_911_/*I*_803_ curve of phlogopite single crystals under γ-ray irradiation at different doses. For the pristine sample, *I*_911_/*I*_803_ is 1.28; for the 1000 kGy-irradiated sample, this value is 2.17. After irradiation, the *I*_911_/*I*_803_ value increased. Assuming the content of Si–O–Al bonds is invariable, the increase in *I*_911_/*I*_803_ implies a reduction in the Si–O bond content, probably indicating that severe radiation damage existed in the high-dose-irradiated sample.

Regardless of the hydroxylation or H_2_O participation in the reaction, the variation in the OH vibration (located at 3710 cm^−1^) is more visible. However, as evident from Fig. 2a, this peak is weak, whereas as evident from Fig. 2b, the baseline is not straight. It seems difficult to accurately calculate the OH vibration. Actually, we observed the FT-IR spectra in the transmission mode; the spectrum is easy to saturate at a low wavenumber and it exhibits shock at a high wavenumber. Simultaneously, the peak is very weak for OH vibrations as the O–H vector is parallel to the direction of photon propagation during measurements. In addition, as the resolution of the vernier caliper is 20 μm and the limit of the eye's resolution, it is difficult to collect and confirm a series of films that are suitable for manual transmission mode experiments. Therefore, it is still difficult to accurately observe OH variations.

Generally, the Raman and FT-ATR spectra reveal slight variations in the chemical structure, which probably indicate H_2_O radiolysis. In most of the cases, the variations in the chemical structure would affect the crystalline structure or macroscopic properties.

### 3.2 Crystalline structure analysis

**Fig. 4:**
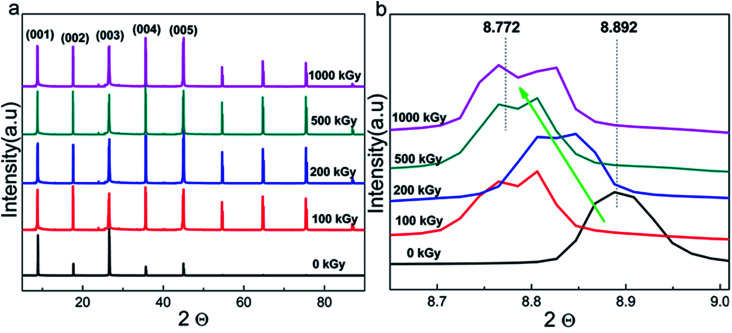
(a) Complete XRD patterns and those of (b) refined (001) lattice plane of phlogopite single crystals under γ-ray irradiation at different doses.

Radiation-induced chemical bond cleavage or new bond formation would affect the crystalline structure, probably resulting in crystal collapse or expansion, affecting *d*. The XRD measurement is effective for characterizing crystalline structure variations. Fig. 4a shows the complete XRD patterns of pristine and irradiated samples. The main lattice planes are assigned by the Jade 5 software according to standard PDF cards. For the pristine sample, there were 5 characteristic lattice planes assigned as (001), (002), (003), (004), and (005) with corresponding 2*θ* values as 8.89°, 17.61°, 26.60°, 35.61°, and 45.05°, respectively. For the irradiated samples, the patterns were similar to that of the pristine sample, except for the several peaks uniformly distributed at 2*θ* values larger than 45° as 54.62°, 64.73°, 75.34°, and 86.73°, respectively.

These additional peaks were probably ascribed to the method utilized for the measurement. As the sample is a thin film when the powder diffraction method is used, the lattice planes might repeat. Finally, these several additional peaks appeared and distributed uniformly. From the macropatterns (Fig. 4a), it is evident that there are no huge variations in the crystalline structure as no special peak is observed except for the several uniform peaks. In other words, no new material or phase formation was observed. Peaks observed at interval positions (say 12, 20, or 29°) would indicate new material or phase formation as different materials have distinct cell parameters. In this case, the material could be completely destroyed, which is extreme and is not expected. Although serious damage was not observed, a partial variation in the refined structure may exist.

Fig. 4b shows the XRD patterns of the (001) lattice plane of pristine and irradiated samples as a representative. After irradiation, the pattern generally shifts toward a lower angle with dose increases to 1000 kGy. For instance, pristine and 1000 kGy-irradiated samples have 2*θ* values of 8.892° and 8.772°, respectively. According to Bragg's formula, *nλ* = 2*d* sin *θ*, for the refined lattice plane and measurement condition, and *n* and *λ* are constant. In this case, a reducing *θ* implies increase in *d.*^51^ In other words, the irradiated sample has larger *d* as compared to that of the pristine sample. This implies expansion, rather than collapse, of the crystal.

To quantificationally describe the expansion level of the (001) lattice plane, *d* was used for comparison. Assuming the aforementioned 2*θ* values, in this case, the ratio of *d* for 1000 kGy-irradiated and pristine samples (*d*_1000_/*d*_0_) is close to 101.4% (*d*_1000_/*d*_0_ = sin *θ*_0_*/*sin *θ*_1000_ = sin 4.446°(8.892/2)/sin 4.386°(8.772/2) ≈ 1.014 × 100% ≈ 101.4%). Simultaneously, *d* has been calculated by the Jade 5 software under the aforementioned *θ* values and *λ* = 0.15418 nm. Therefore, *d*_0_ and *d*_1000_ are 9.9372 ± 0.003 and 10.071 ± 0.015 Å, respectively. In this case, *d*_1000_/*d*_0_ = 10.071/9.9372 ≈ 1.01346 × 100% ≈ 101.3%, which is close to the ratio calculated by using sin *θ*. This means that *d* of the (001) lattice plane increased by 1% under 1000 kGy γ-ray irradiation.

After 1000 kGy γ-ray irradiation, *d* of the (001) lattice plane increased by 1%. This variation may be tiny as compared to that during ion irradiation, as ions such as He^2+^ and Au^3+^ have considerable volume and charge, which would easily induce the movement of atoms in the lattice and interlayer.^52^ However, this variation is visible and probably obvious for γ-ray irradiation and this phenomenon is interesting. For clay used in the disposal of HLRW, it may remain irradiated for tens of thousands of years or even longer.^53^ In that case, the accumulated dose may be very high. In this work, the 1000 kGy irradiation covers 500 years of irradiation.^31^ Here, *d* of the (001) lattice plane increased by 1%. If the dose covers the irradiation level for a longer time such as 1000 or 10 000 years, *d* may vary more considerably. Simultaneously, a small variation in the crystalline structure might have an obvious effect on the retention capability and mechanical properties,^54^ affecting the ultimate security of the disposal project. This 1% increase is close to 0.13 ± 0.01 Å (*d*_1000_ − *d*_0_ = 10.071(±0.015) − 9.9372(±0.003) ≈ 0.13(±0.01) Å), which seems small. Nevertheless, the bond lengths of Si–O and Al–O are close to 1.65 and 2 Å.^55^ For phlogopites, the linkage between the TO_4_ tetrahedron and MgO_6_ octahedron is the O atom. In other words, the Mg–O–Si or Mg–O–Al bonds are not parallel to the *z* direction. Even the Mg–O–Si bond is completely destroyed and new chemical bonds are formed; the distance of the T–O–T structure in the *z* direction cannot increase by a large amount as the bond length of the Si–O/Mg–O is just several angstroms and the amount of Si–O bonds in the tetrahedron is lean. Taking these factors into consideration, the 1% increase in *d* of the (001) lattice plane is obvious and tremendous. In other words, the crystalline structure changed considerably under a high dose of irradiation. The increase in *d* is probably due to the formation of additional chemical bonds or chemical reactions.

No other matter contacted the sample except for air during the irradiation process. The additional chemical bonds pulled in could be probably ascribed to H_2_O radiolysis as H_2_O generally exists in the interlayer or on the clay surface.^16^ When H_2_O participates in this reaction, its content would decrease.

Generally, the XRD measurements show an expansion of phlogopites under higher doses of γ-ray irradiation. In other words, additional groups were introduced.

### 3.3 Thermal stability analysis

In earlier reports,^25,38,41,49^ thermally induced dehydroxylation would not occur at temperatures below 500 °C Under this assumption, mass variations at temperatures lower than 500 °C could be ascribed to the volatilization of H_2_O originally existing other than that during the dehydroxylation process during measurements. In this case, the TGA measurement could probably reflect the variations in the H_2_O content.

**Fig. 5:**
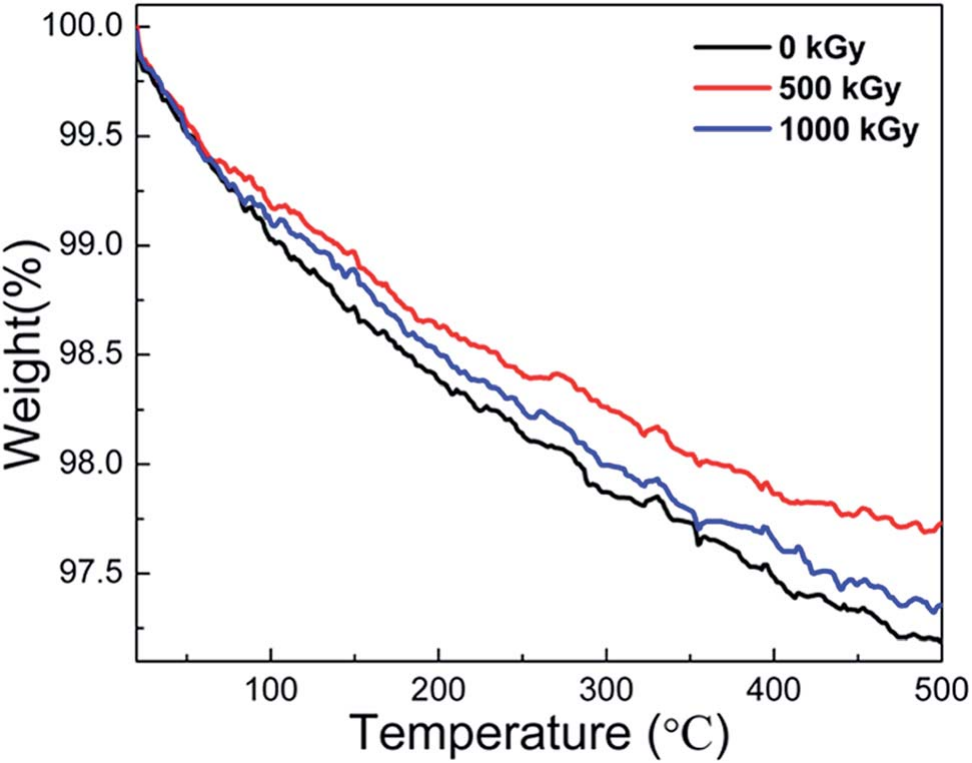
TGA curves of pristine, 500 kGy-irradiated, and 1000 kGy-irradiated phlogopites.

Fig. 5 shows the TGA curves of pristine, 500 kGy-irradiated, and 1000 kGy-irradiated samples, respectively. All the curves are similar. When the temperature increases to 500 °C, the mass reduces marginally. For instance, the mass of pristine, 500 kGy-irradiated, and 1000 kGy-irradiated samples decreases to 97.2, 97.7, and 97.3%, respectively. From the curves, no sharp decline was evident, indicating that no intense processes (volatilization of organic compounds, dehydroxylation, and decomposition) occurred during the measurement process. Assuming the sample is pure without any impurity except for H_2_O, the slight reduction in this case could be ascribed to the volatilization of H_2_O as H_2_O generally existed in clay^16,46^ and can be evaporated at 50–500 °C.^16,56^

We assume that the volatilization of H_2_O is in line with its content. Simultaneously, we assumed that the content of H_2_O within the sample before irradiation was the same. Therefore, the mass reduction in the TGA curve is lined with its content. The pristine sample has the largest mass reduction at 2.8% (100–97.2% = 2.8%). The 500 kGy- and 1000 kGy-irradiated samples have mass reductions of 2.3% (100–97.7% = 2.3%) and 2.7% (100–97.3% = 2.7%), respectively. In other words, the H_2_O amount in pristine, 500 kGy- and 1000 kGy-irradiated samples could be concluded to be 2.8, 2.3, and 2.7%, respectively. This implies that the H_2_O amount in the irradiated sample is lower than that of the pristine sample, showing declined H_2_O amounts after irradiation.

As the γ-ray irradiation is a cold irradiation mode as compared to electron-beam irradiation,^57^ the radiation-induced temperature variations within the sample during the irradiation procedure can be ignored. In this case, the volatilization of H_2_O induced by temperature increase related to irradiation during the irradiation procedure can be ignored. In this case, the variation in the H_2_O content after irradiation can be ascribed to its reaction with the sample matrix.

To our knowledge, H_2_O can easily undergo radiolysis under the influence of γ-ray or electron-beam irradiation.^27,53^ After irradiation, H˙ and HO˙ radicals are generated,^13,16,17,20^ which are reaction-active. They can easily react with the sample matrix, such as the tetrahedron or octahedron sheets.^45^ After these reactions, extra H atoms or OH groups are introduced, which would enlarge the tetrahedron or octahedron units, increasing *d* as the expansion can easily occur along the *z* direction as compared to those along the *x* and *y* directions.^49^ This assumption could probably explain the increase in *d* after irradiation, as confirmed by the XRD measurements. In some cases, the introduction of additional H atoms or OH groups could be attributed to the (Si–O–Mg/Si–O–Al) link breakage. As this link breaks, the content of Si–O stretching (Si atom to non-bridge O) decreases. Finally, the ratio of Al–O–Si/Si–O could increase. This assumption could indirectly certify the FT-ATR results. In addition, the introduction of additional H atoms or OH groups would increase the OH content. In this case, if we ascribe the peak at 143 cm^−1^ in RS to OH vibrations, the relative intensity of this peak should increase. In reality, the relative intensity of this peak increased after 200 and 500 kGy irradiation. This assumption could indirectly certify the RS results.

Except for the introduction of additional H atoms or OH groups, the framework would break,^13^ which would collapse the tetrahedral or octahedral units, minifying the volume parameters (*e.g.*, *d*). During the irradiation process, these two procedures occur synchronously. Finally, the variation in *d* is a combination of the collapse induced by the framework breaking and expansion induced by the introduction of additional H atoms or OH groups. From the XRD results, it is evident that *d* increases after irradiation. This indicates more expansion occurred in the sample than collapse, and the H_2_O within the sample matrix probably exhibited a key role toward radiation damage.

Generally, the TGA measurements show reduced H_2_O content in the irradiated sample. The variation in the H_2_O content was probably due to its radiolysis, which would result in the introduction of additional chemical bonds in the framework and increasing *d*. In this case, the TGA result can certify the explanation that the expansion of the crystal is suitable, verifying the RS and FT-ATR results. Simultaneously, there is no obvious variation in the thermal stability, regardless of irradiation.

### 3.4 Surface hydrophilicity and morphology

**Fig. 6:**
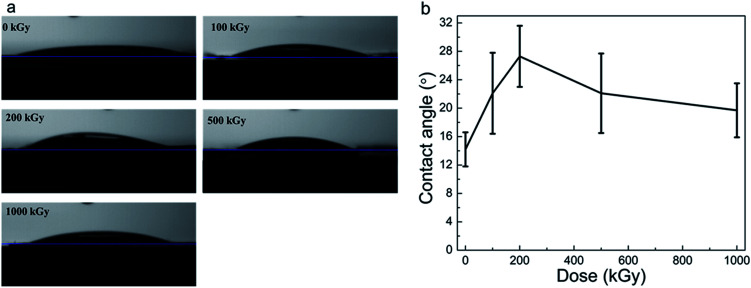
(a) Optical images of water droplets on the sample surface and (b) static CAs of phlogopite single crystals under γ-ray irradiation at different doses.

Radiation-induced chemical structure variations, defects, or H_2_O radiolysis would affect surface wettability.^58^ Static CA experiments are effective in characterizing surface hydrophilicity.^34,35^Fig. 6a and b show the optical images of water droplets on the sample surface and the static CAs of phlogopites under γ-ray irradiation of 1000 kGy. From Fig. 6a, it is evident that the droplet on the pristine sample almost spreads out completely, exhibiting excellent hydrophilicity. For the irradiated sample, the sprawl of the droplet is similar to that of the pristine sample, showing worthwhile hydrophilicity. Nevertheless, a partial variation probably existed particularly for the 200 kGy-irradiated sample.

From Fig. 6b, it is evident that the pristine sample has the smallest CA of approximately 15°. The irradiated samples have larger and different CAs. For instance, the CA increased to approximately 28° with the dose increase to 200 kGy and then decreased to approximately 20° as the dose continued to increase to 1000 kGy. Although the CA varied, the variation range is less than 15°, which is small. This small range of CA variation indicates a marginal change in the hydrophilicity characteristic. Generally, the irradiated samples almost have no obvious changes in the CA, showing worthwhile hydrophilicity.

Although the CA did not show obvious changes, its value increased at low doses and then decreased at doses higher than 200 kGy. This could be explained as follows. For the low-dose-irradiated sample (≤200 kGy), the degree of destruction (breakage of chemical bonds within TO_4_ tetrahedra like Si–O bonds and partial Si–OH bonds on the surface or edges) would increase with the dose, resulting in reduced hydrophilicity. Simultaneously, the partial Mg–OH bonds would break,^17,27^ also reducing the hydrophilicity. However, from the RS and FT-ATR analysis results, it is evident that the absence of dehydroxylation was assured. From the XRD and TGA analysis results, it seems that partial H_2_O underwent radiolysis and additional OH groups were introduced. In this case, hydrophilicity should increase and CA should reduce. Nevertheless, the obtained result seems inconsistent with our expectations. This is probably because of the structure of the material itself. This is because the introduced OH groups probably mainly existed in the interior of the T–O–T sheet (certified indirectly by the XRD results for the expansion of the (001) lattice plane). In this case, the additional OH groups probably did not exist on the sample surface itself but existed in the octahedron sheets or the in-plane of the tetrahedron sheets. However, hydrophilicity probably relies on the surface structure, which mainly contains tetrahedron structures and interlayer ions (*e.g.*, K^+^). From Fig. 6a, it is evident that the surface of the pristine sample exhibits excellent hydrophilicity because of the hydrophilic Si–O or Si–OH bonds.^59^ After irradiation, a partial surface structure undergoes destruction;^41^ the degree of destruction would increase with the dose. In this case, the destruction may have an increased impact on the surface hydrophilicity than that by the introduced OH groups. Therefore, hydrophilicity could decline under a lower dose of irradiation.

With dose increase, the radiolysis of H_2_O becomes serious; partial H˙ and HO˙ radicals might migrate toward the sample surface, introducing polar groups on the tetrahedron structure, increasing the hydrophilicity. Simultaneously, a partial breakdown of the Si–OH groups or defects on the surface or edges could be recovered,^60,61^ also elevating the hydrophilicity. From the structure, however, it is evident that Si–OH groups should not exist on the surface, but partial Si–OH groups generally exist on silicate^2^ or oxide surfaces such as SiO_2_ particles,^62–64^ glass, *etc.* In addition, defects introduced by irradiation might increase surface roughness. The increased roughness might enlarge the contact area, strengthening the interfacial force within the sample matrix and H_2_O, increasing wettability. For the high-dose-irradiated sample, the aforementioned variations could be obvious. Therefore, a spinodal was observed in the CA results. These assumptions could probably explain the CA decrease at doses higher than 200 kGy.

All the previous explanations were on account of the microscopic structure at the molecular or atomic levels, while the macroscopic morphology variations would also affect the obtained CA. For a hydrophilic material, according to the Wenzel model, the larger the roughness, the smaller is the CA. In this case, if there were partial grooves or cracks on the sample surface induced by an artificial factor, the CA would sufficiently decrease. This might exceed the effect induced by irradiation on the surface properties. In this case, all the aforementioned explanations were probably inappropriate. Therefore, it is essential to observe the macroscopic morphology of the sample surface. From Fig. 6b, it is evident that there was a spinodal at 200 kGy. For a comparison, pristine and 1000 kGy-irradiated samples were also observed.

**Fig. 7:**
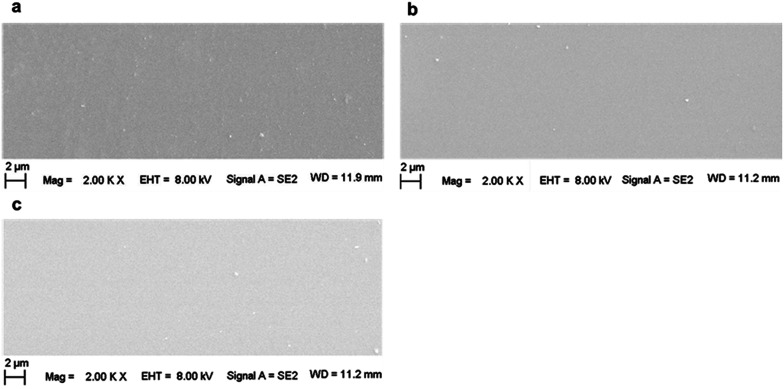
SEM images of the surfaces of (a) pristine, (b) 200 kGy-irradiated, and (c) 1000 kGy-irradiated samples.

Fig. 7 shows the SEM images of the surface of (a) pristine, (b) 200 kGy-irradiated, and (c) 1000 kGy-irradiated samples, respectively. Generally, the surface morphologies are smooth, showing no obvious grooves or cracks. This indicates that the films prepared for the CA experiments were smooth and no obvious grooves or cracks existed that could be induced by an artificial factor. In addition, the smooth surface also indicates that the irradiation almost had no obvious effect on the macroscopic morphology. In this case, we could eliminate the effect of any artificial factors on the obtained CA. In other words, the CA variation was not derived from the sample preparation, but it was obtained from the microscopic structural variations. This observation could certify the fact that the previous explanation is suitable. From a careful inspection of Fig. 7b and c, it seems that the surface of the 1000 kGy-irradiated sample is smoother than that of the 200 kGy-irradiated sample. In other words, the surface of the 200 kGy-irradiated sample is rougher than that of the 1000 kGy-irradiated sample. Assuming there was no obvious difference within the microscopic structures of the 200- and 1000 kGy-irradiated samples, in this case, the former should have better hydrophilicity than the latter. In other words, the 200 kGy-irradiated sample should have smaller CA as compared to the 1000 kGy-irradiated sample. Nevertheless, a contrary result was obtained. The 1000 kGy-irradiated sample had a smaller CA as compared to the 200 kGy-irradiated sample. This further indicates microscopic structural variations. The tiny variations within the surface morphologies of the 200- and 1000 kGy-irradiated samples could be probably ascribed to irradiation. As the collision of γ photons with the surface atoms is random, partial low-lying sites can be elevated by the additional chemical bond formation and the partially raised sites can be cut down by breakage. Then, the surface seems to be smoother under a higher dose of irradiation. This procedure should be certified. Nevertheless, this tiny variation probably exceeds the resolution of current SEM technology. In reality, an earlier study has reported the effect of irradiation on the surface morphology of mica. Even under ^197^Au (14.0 MeV/*n*) ion irradiation for 30 s, the defect along the *z* direction for muscovite is several nanometers.^65^ For γ-ray irradiation, the variation would be smaller, which would even exceed the resolution of AFM technology. Therefore, this procedure for roughness variation was not certified. Generally, from the SEM experiments, it can be concluded that CA variations could be approximately ascribed to the variation in the intrinsic structure and not to any artificial factors.

Actually, hydrophilicity variations have a complex mechanism, including the destruction of TO_4_ tetrahedra and MO_6_ octahedra, introduction of additional OH related to H_2_O radiolysis, and roughness variations. These processes occurred synchronously and might have contrary effects on surface hydrophilicity. Finally, a slight variation within the hydrophilicity was observed.

From the CA, SEM, XRD, and TGA experiments, it seems that radiation-induced destruction and H_2_O radiolysis had a crucial impact on the surface wettability of phlogopites. For the low-dose-irradiated sample, radiation-induced destruction is the dominant factor to affect hydrophilicity, while for the high-dose-irradiated sample, H_2_O radiolysis and its reaction with the surface structure predominantly affects the hydrophilicity.

In general, the CA and SEM experiments indicate that γ-ray irradiation with doses up to 1000 kGy has no obvious impact on the surface properties of phlogopites and a low dose of irradiation could reduce surface hydrophilicity.

### 3.5 Mechanism illustration

In the abovementioned sections, radiation stability within the chemical/crystalline structure and surface hydrophilicity were evaluated, and their mechanisms were explored. Nevertheless, these mechanisms are explained loosely. To have a clear understanding, it is illustrated below. Generally, the main mechanism in radiation damage involves framework destruction and H_2_O radiolysis. Upon irradiation, the chemical bonds within the tetrahedron sheet and linkage between the tetrahedron and octahedron break. In this case, the relative intensity of Si–O stretching and hydrophilicity declined at a lower dose. Except for the framework breakage, partial H_2_O underwent radiolysis. The radiolysis products (H˙ and HO˙ radicals) are reaction-active. They react with the tetrahedra or the linkage between the tetrahedron and octahedron sheets. Finally, additional OH groups were introduced, enlarging *d* and expanding the lattice plane, thereby recovering the hydrophilicity. This phenomenon is more obvious at higher doses. During irradiation, these two procedures occurred synchronously, and H_2_O radiolysis seems more important.

**Fig. 8:**
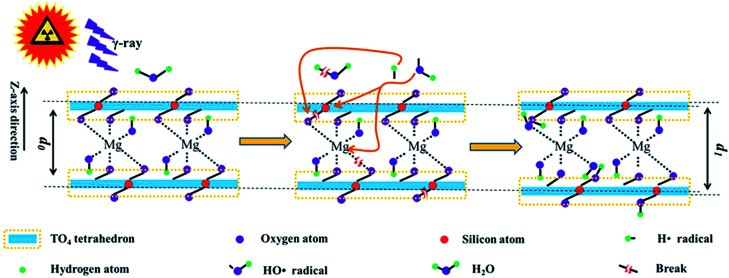
Schematic of irradiation on the phlogopite structure; *d*_0_, interlayer space for pristine sample; *d*_1_, interlayer space for sample after irradiation.

The reaction equation can be used to make this procedure more visible. As a tetrahedron sheet mainly contains Si–O–Si or Si–O–Al bonds, ≡Si–O–Si≡ could represent the tetrahedron sheet. Simultaneously, the linkage between the tetrahedral and octahedral sheets is through O atoms, namely, Si–O–Mg or Al–O–Mg bond. In other words, *d* mainly reflects the size of Si–O–Mg–O–Si along the *z* direction. Therefore, ≡Si–O–Mg–O–Si≡ could represent the T–O–T structure along the *z* direction. Under these assumptions, the main procedure can be described using eqn (1)–(9) and as shown in Fig. 8.

Eqn (1)–(5) are the probable reactions originally induced by γ-ray irradiation.

1 ≡Si–O–Si≡ → ≡Si˙ + ·O–Si≡

2 ≡Si–OH → ≡Si˙ + HO˙

3 ≡Si–O–Mg–O–Si≡ → ≡Si˙ + ·O–Mg–O–Si≡

4 ≡Si–O–Mg–O–Si≡ → ≡Si–O˙ + ·Mg–O–Si≡

5 H_2_O → H˙ + HO˙

Eqn (6)–(9) are the probable reactions between the radiolysis products.

6 ≡Si˙ + HO˙ → ≡Si–OH

7 ≡Si–O˙ + H˙ → ≡Si–OH

8 H˙ + ·O–Mg–O–Si≡ → HO–Mg–O–Si≡

9 HO˙ + ·Mg–O–Si≡ → HO–Mg–O–Si≡

Generally, eqn (1) and (2) describe the tetrahedral destruction, which could probably illustrate the hydrophilicity decline under a lower dose of irradiation. Eqn (3) and (4) describe the link breakage between the tetrahedron and octahedron sheets. Eqn (5) describes H_2_O radiolysis, which could illustrate the decline in H_2_O amount. Eqn (6)–(9) describe the introduction of OH, which could probably illustrate hydrophilicity recovery at higher doses and increase in *d* under irradiation.

## 4. Conclusions

The radiation stability of phlogopite single crystals under γ-ray irradiation was evaluated with regard to their chemical/crystalline structures and macroscopic properties. Upon irradiation, the chemical structure, thermal stability, and surface morphology changed marginally, while the crystalline structure varied obviously. Under 1000 kGy irradiation, the *d* value of the (001) lattice plane increased by more than 1%, showing expansion. In addition, the surface hydrophilicity declined at a lower dose. From the chemical structure, surface morphology, and thermal stability analyses, it is evident that phlogopite single crystals seem radiation-resistant. Nevertheless, the 1% expansion of the (001) lattice plane is huge and this phenomenon is interesting. Normally, any crystalline structure variation has considerable impact on its mechanical properties. In this case, it can be concluded that phlogopites are sensitive toward γ-ray irradiation with regard to their crystalline structure and have poor radiation resistance. Simultaneously, the mechanisms of the radiation effects were explored. Generally, the radiation stability of phlogopite single crystals under γ-ray irradiation involves framework destruction and environmental conditions. Upon irradiation, the framework within the tetrahedron and octahedron sheets suffered breakage, declining the hydrophilicity characteristics. Except for framework destruction, H_2_O underwent radiolysis, and additional H atoms or OH groups were introduced. During the irradiation procedure, these two procedures occurred synchronously, and H_2_O radiolysis seems more important. Finally, the crystal structure underwent expansion, and the content of H_2_O and relative content of Si–O stretching declined, thereby recovering the hydrophilicity. This finding is meaningful to explore the mechanism of radiation damage and to evaluate the radiation stability of the matrix of clays used in the disposal of HLRW in practice.

Some drawbacks of this work are that the level of dehydroxylation was not clearly evaluated, H_2_O radiolysis products were not clearly observed, and explanations of surface property variations and mechanism explorations were speculated. These drawbacks are critical for realizing the mechanism of radiation damage within phlogopites and the matrix of clay used in practice, which needs comprehensive research efforts. Generally, this work can be beneficial in investigating the mechanism of radiation damage of clays.

## Conflicts of interest

There are no conflicts to declare.

## Supplementary Material
